# Conjunctivitis induced by a red bodied mite, *Neotrombicula autumnalis*

**DOI:** 10.1051/parasite/2013025

**Published:** 2013-07-04

**Authors:** Benjamin J. Parcell, Graeme Sharpe, Brian Jones, Claire L. Alexander

**Affiliations:** 1 Department of Microbiology, Ninewells Hospital Dundee DD1 9SY United Kingdom; 2 Department of Ophthalmology, Ninewells Hospital Dundee DD1 9SY United Kingdom; 3 Department of Microbiology, Glasgow Royal Infirmary Glasgow G4 0SF United Kingdom; 4 Scottish Parasite Diagnostic and Reference Laboratory, Stobhill Hospital Glasgow G21 3UW United Kingdom

**Keywords:** *Neotrombicula autumnalis*, Mite, Conjunctivitis, Trombiculosis, Case report

## Abstract

This is a description of an unusual case of conjunctivitis caused by a trombiculid red mite, *Neotrombicula autumnalis*. The patient’s condition improved only after its removal and with application of carbomer gel eye drops. There have been reports of increasing numbers of severe cases of trombiculosis over the last 15 years particularly in Germany and a number of cases have also been reported in the United Kingdom. Cases where trombiculid larvae feed on any region of the head or face of humans are unknown. In addition it is most likely the patient acquired the infection from her pet cat and this is the first description of acquisition from this animal.

## Abstract (fr)

Ceci est la description d’un cas inhabituel de conjonctivite causée par un acarien Trombiculidae, *Neotrombicula autumnalis*. La condition de la patiente ne s’est améliorée qu’après extirpation de l’animal et application de gouttes pour de gel pour les yeux au carbomer. Un nombre accru de cas sévères de trombiculose a été rapporté ces quinze dernières années d’Allemagne et des cas ont aussi été rapportés du Royaume Uni. Les cas où les aoûtats se nourrissent sur une région de la tête ou de la face sont inconnus. De plus, il est très probable que la patiente a acquis l’infection de son chat, et ceci est la première description d’une transmission depuis cet animal.

## Introduction

One of the most common red bodied mites in Europe is *Neotrombicula autumnalis* which is known by many names such as the harvest mite, *lepte autumnal* and aoutat [1, 2]. There exist over 1,200 species of trombiculid mite found widely distributed in many countries of which approximately 50 can cause disease in humans or animals. They belong to the family Trombiculidae [1]. Clinical presentations of *Neotrombicula autumnalis* include pruritic dermatitis described as trombiculosis or Scrub itch. Previously, patients were rarely referred for dermatologist review unless symptoms were severe. Over the last 15 years, cases of severe trombiculosis have increased in Western Germany and in the United Kingdom [2, 3].

## Case report

**Figure 1.**
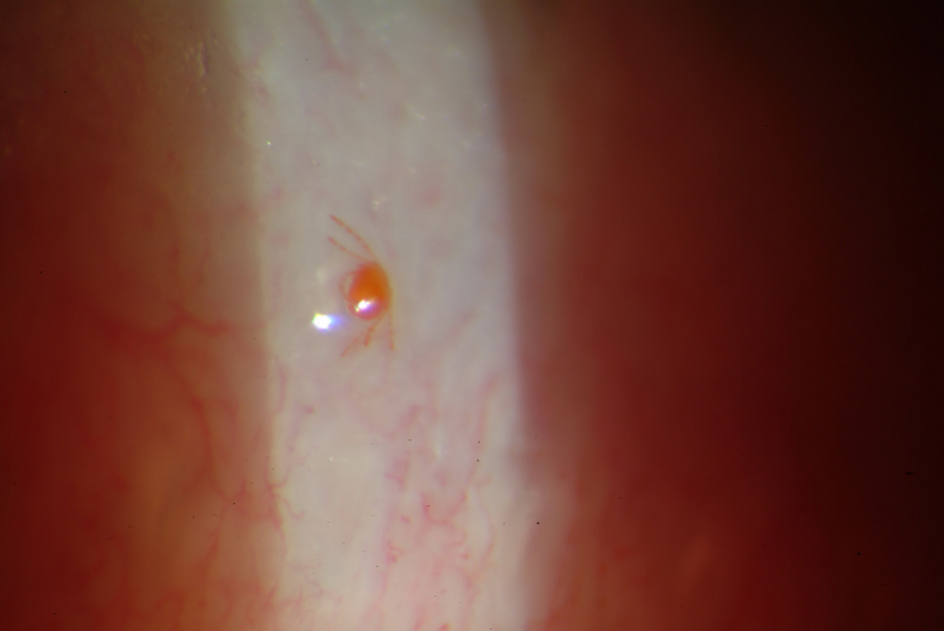
*Neotrombicula autumnalis* mite in contact with the left upper eyelid margin.

A 72-year-old female from Perthshire, Scotland, United Kingdom presented to the Outpatient Ophthalmology Clinic with a two-week history of a painful, gritty, red left eye which failed to improve with a liquid paraffin eye ointment, Lacri-Lube. On examination, her conjunctiva was found to be mildly red and she had normal visual acuity. On close inspection, a live mite was identified in contact with the left upper eyelid margin. The patient’s past medical history included left eye cataract surgery twelve weeks prior, not thought to be of significance to her current presentation and from which she had made a full recovery. She reported no history of travel or hill walking, lived independently and kept a pet cat. The mite was photographed and removed without local anaesthetic. Photographic images were sent to the Scottish Parasite Diagnostic and Reference Laboratory, Stobhill Hospital (Figure 1). On examination the mite’s mouthparts were attached and inserted into the tissue of the host. The mite was identified as the six-legged larva of a red bodied trombiculid red mite, *Neotrombicula autumnalis* which is a common ectoparasite on mammals in the United Kingdom. After removal of the mite, the patient was treated with Carbomer gel eye drops, namely Viscotears, and her symptoms resolved.

## Discussion

Mite larvae, also known as chiggers, are most active from May to October [3]. They hatch from eggs in the soil after 10 days and climb onto grass or leaves and are barely visible without magnification. The six-legged larvae may be white to bright red in colour and are 0.25 mm long [1]. It is the larvae that are animal and human ectoparasites and they have a unique way of feeding on human hosts [4]. The larvae do not burrow but tend to attach in clusters [3]. They pierce the skin using sharp mouthparts (chelicerae) and tissue dissolving saliva is injected via a straw-like hollow tube (hypostome) [4]. The larvae usually feed for approximately 2–10 days or longer and then fall to the soil. Here, they develop into eight-legged nymphae then undergo another quiescent phase to become adults [1, 4, 5]. Adult and nymphal mites are soil dwelling predators which feed on other arthropods and their eggs [2]. Feeding can result in a pruritic dermatitis described as trombiculosis presenting with red macules, or wheals with papules 3–6 h after exposure [1]. In severe cases papulovesicles with regional adenitis can occur [3]. Distribution of the lesions depends on the type of clothing worn and area of invasion by the mites [2, 3]. Usually it is the ankles that are affected although any area exposed can result in dermatitis. There have been reports of severe cases of trombiculosis over the last 15 years particularly in Western Germany and in the United Kingdom [2, 3, 6, 7]. Trombiculosis is well recognised in areas where the harvest mite is common and cases are rarely referred to local dermatologists unless severe [3]. Treatment recommendations for trombiculosis are mostly supportive and have included topical agents such as methyl alcohol or camphor [3]. Even common household vinegar (5% acetic acid) has been described in one case as an effective agent to reduce infestations [7]. Gamma benzene hexachloride, oral antihistamines, topical steroids, vacuuming and washing clothes at 55 °C have been advised [3–5]. As most cases are transmitted when humans or animals come into contact with vegetation or soil that is infested, measures such as wearing protective clothing, avoiding walking through long grass, soaking socks and trouser legs with benzyl benzoate, dimethyl phthalate (DPM) or diethyltoluamide insect repellents (DEET) have also been recommended as ways to prevent infestation. Permethrin repellent has been used in the past however the active ingredient is no longer available for this purpose in the European Union [1, 3].

Trombiculid mites, in particular those of the genus *Leptotrombidium*, are known to be vectors of Tsutsugamushi disease (“scrub typhus”) in the South Pacific, Oceania and Asia [6]. *Neotrombicula autumnalis* larvae were not believed to transmit any infections however, a recent study suggests that they may have the potential to transmit, via transstadial and transovarial routes, the bacterium *Borrelia burgdorferi*, the cause of human Lyme disease [6]. In addition, another recent study has suggested that *Neotrombicula autumnalis* may be a carrier of *Anaplasma phagocytophilum* (formerly *Ehrlichia phagocytophila*) which causes human granulocytic anaplasmosis. This was previously believed to be transmitted by ticks only [5, 8]. Both these infections can cause significant morbidity and fatalities. The numbers of these infections have also increased overall over the last ten years [9,10]. Further studies are needed to demonstrate transmission of these infections to hosts as diagnostic methods and treatment are available.

We believe that the patient acquired the mite from her cat as she had no history of travel, hill walking, gardening or contact with long grass or vegetation. This is an unusual route of infection and only one previous publication has described trombiculosis acquired from close human contact with pet dogs [5]. Disease of the head or face is unknown and this is the first reported case of conjunctivitis caused by a red bodied mite, demonstrating the importance of this differential diagnosis for those living in geographical areas in which the mite is particularly abundant.
